# Imipramine alters the sterol profile in *Leishmania amazonensis* and increases its sensitivity to miconazole

**DOI:** 10.1186/s13071-016-1467-8

**Published:** 2016-03-31

**Authors:** Valter Viana Andrade-Neto, Thaís Martins Pereira, Marilene do Canto-Cavalheiro, Eduardo Caio Torres-Santos

**Affiliations:** Laboratório de Bioquímica de Tripanosomatídeos, Instituto Oswaldo Cruz, FIOCRUZ, Avenida Brasil 4365, Manguinhos, Rio de Janeiro Brazil

**Keywords:** *Leishmania*, Imipramine, Sterols

## Abstract

**Background:**

Imipramine, a tricyclic antidepressant widely used clinically, has other pharmacological effects, such as antileishmanial activity. Tricyclic antidepressants interact with lipid bilayers, and some studies have shown that imipramine inhibits methyltransferases. *Leishmania* spp. produces compounds with an ergostane skeleton instead of a cholesterol skeleton, and the inhibition of enzymes of the sterol biosynthesis pathway is an interesting therapeutic target. Among these enzymes, C-24 methyltransferase has been suggested to play an essential role, as its inhibition kills the parasites. In this context, we investigated whether imipramine alters the biosynthesis of sterols in *L. amazonensis* and evaluated the efficacy of imipramine alone and in combination with miconazole, a classical inhibitor of another step in this pathway.

**Methods:**

To analyze the interference of imipramine with sterol metabolism, promastigotes of *L. amazonensis* were cultured with medium alone, 15 or 30 μM imipramine or 4 μM miconazole, and their lipids were extracted with methanol/chloroform/water (1:0.5:0.4 v/v) and analyzed by GC/MS. To assess the antileishmanial activity of the treatments, promastigotes of *L. amazonensis* were incubated with various concentrations of imipramine up to 100 μM and up to 24 μM miconazole. Promastigotes were also treated with the combination of imipramine and miconazole at concentrations up to 12.5 μM of imipramine and 24 μM of miconazole. Parasite growth was evaluated by the MTT assay. The fractional inhibitory concentration index (FICI) was calculated to determine whether there were synergistic effects. Peritoneal macrophages with and without *L. amazonensis* infection were treated with miconazole (0 – 16 μM) or imipramine (0 to 50 μM) for 72 hours. For assays of the combined treatment in amastigotes, the concentration of imipramine was fixed at 12.5 μM and various concentrations of miconazole were used up to 16 μM. The infection rate was determined by counting the infected macrophages under a light microscope.

**Findings:**

Promastigotes treated with imipramine accumulated cholesta-5,7,22-trien-3β-ol and cholesta-7-24-dien- 3β-ol, sterols that normally increase after treatment with classical inhibitors of C-24 methyltransferase. The IC_50_ of miconazole in promastigotes decreased when it was used in combination with imipramine, resulting in an additive effect, with a FICI value of 0.83. Imipramine also showed activity against intracellular amastigotes and enhanced the activity of miconazole, without apparent toxicity to the host cells.

**Conclusions:**

Imipramine was confirmed to have antileishmanial activity in both forms of the parasite, affecting the sterol biosynthesis of the organisms. Using imipramine in combination with azoles may be advantageous for the treatment of leishmaniasis.

## Background

Leishmaniasis is a disease caused by parasites of the genus *Leishmania*, unicellular eukaryotes belonging to class Kinetoplastea and family Trypanosomatidae. *Leishmania* spp. are transmitted by more than 20 species of sandflies of the genera *Phlebotomus* and *Lutzomyia* [1]*.* Leishmaniasis is a non-contagious infectious disease that can affect the skin and mucous membranes (cutaneous leishmaniasis) or internal organs (visceral leishmaniasis) [2, 3]. Endemic transmission of leishmaniasis is known to occur in 98 countries on five continents. It has been estimated that there were more than 58,000 cases of visceral leishmaniasis and 220,000 cases of cutaneous leishmaniasis [4].

Since the 1940s, the main treatment for leishmaniasis has included antimony derivatives that are commercially available in two formulations, N - methylglucamine antimoniate (meglumine antimoniate, Glucantime®) and sodium stibogluconate (Pentostam®) [5]. These drugs have many toxic effects, including cardiac, hepatic, pancreatic and renal toxicity, and should be used with caution and with clinical and laboratory monitoring in patients with heart or liver disease [6]. The efficiency of antimony may vary, and treatment protocols are determined depending on the region. Many cases of resistance have been reported, making treatment difficult [7]. Miltefosine, the only oral treatment, has been prescribed in India for visceral leishmaniasis for more than a decade and was recently approved by the FDA for all forms of this disease, but some concerns about resistance and teratogenicity have been raised [8, 9]. Other drugs, such as pentamidine and amphotericin B, may be used if resistance to the first-line treatment occurs, although these have major toxicity [5]. Furthermore, no vaccine for human use has been developed to date, although some studies have been carried out, such as a study of the protective role of serine proteases in the parasite [10]. Thus, there is a need to search for new strategies to control leishmaniasis.

Imipramine, a tricyclic antidepressant widely used in the clinical setting, is used in the treatment of severe chronic depression because it inhibits5-hydroxytryptamine (serotonin) and norepinephrine reuptake [11]. Beyond its actions on the central nervous system, imipramine also has other biological effects, such as immunosuppressive properties through the modulation of inflammatory cytokine production and the induction of autophagic death in tumor cells [12, 13]. The antileishmanial effects of imipramine and its analogs were first demonstrated in the 1980s in promastigotes and amastigotes of *L. donovani* and *L. major* [14]. Imipramine also exhibited activity against antimony-resistant intracellular amastigotes without affecting the host cells. Additionally, oral treatment with imipramine reduced the parasite burden of visceral leishmaniasis in hamsters [15].

Tricyclic antidepressants have high affinity for the phosphatidylcholine and phosphatidylethanolamine in lipid membranes, causing changes in lipid-protein interactions [16]. Another interesting feature of imipramine is its effect on methyltransferases. Imipramine has been described to inhibit the activity of catechol-O-methyltransferase (COMT), DNA methyltransferase and the methylation of phospholipids in the plasma membrane [17, 18].

The sterol biosynthesis pathway of trypanosomatids has diverged from that in mammalians such that these parasites produce ergosterol derivatives instead of cholesterol [19]. It is assumed that this biosynthetic pathway is essential in *Leishmania* because pharmacological inhibition of different steps results in the death of the parasite [20–25]. Methylation by C-24methyltranferase is one of final steps of sterol biosynthesis in *Leishmania,* and this reaction does not occur in mammalians. Inhibition of this enzyme by azasterols causes the accumulation of cholestane derivatives and leads to parasite death [26].

Here we investigated the effects of imipramine on the sterol biosynthesis of *L. amazonensis* and its leishmanicidal activity in combination with miconazole, a well-known inhibitor of this pathway.

## Methods

### Drugs

Miconazole and imipramine were provided by Sigma–Aldrich (St. Louis, USA) and stored at -20^o^ C in DMSO (Sigma-Aldrich) stock solution (10 mM).

### Maintenance and cultivation of parasites

Promastigotes of *Leishmania amazonensis* (strain MHOM/BR/77/LTB0016) were maintained at 26 °C in RPMI medium (Sigma- Aldrich) supplemented with 10 % fetal bovine serum (FBS), 100 μg/ml streptomycin, 100 U/ml penicillin and 5 mg/ml hemine. Subcultures were performed twice a week until the tenth passage. Afterwards, old cultures were discarded and fresh parasites were obtained from infected BALB/c mice.

### Antipromastigote activity

Promastigotes of *L. amazonensis* were maintained in cell culture flasks at 26 °C in RPMI medium without phenol red (Sigma- Aldrich) supplemented as described above. Experiments were performed on promastigotes in 96-well plates, with an initial inoculum of 1.0 × 10^6^cells/ml and compound concentrations of up to 24 μM of miconazole and to 100 μM of imipramine. In the combination experiments, the concentrations were up to 24 μM of miconazole and 12.5 μM of imipramine. Plates were incubated at 26 °C for 72 hours. After this period, parasite growth was evaluated by adding 10 % tetrazolium salt (MTT, Sigma-Aldrich) (5 mg/ml) per well. The plates were incubated at 26 °C for a further hour, and formazan crystals were dissolved by adding 80 μl of DMSO to each well. The reaction was analyzed using a spectrophotometer at 570 nm wavelength. IC_50_ values were obtained by non-linear regression using the GraphPad Prism 6 software (GraphPad Software Inc., La Jolla, USA). The fractional inhibitory concentration (FIC) determined to analyze whether the combination treatments were synergistic was calculated as follows: FIC = IC_50_ of drug A in combination/IC_50_ of drug A alone + IC_50_ of drug B in combination/IC_50_ of drug B alone. The arithmetic mean of the FICs obtained for each concentration was interpreted according to published guidelines: synergy (FIC ≤ 0.5), antagonism (FIC ≥ 4.0) and additive effect (no interaction) (0.5 < FIC < 4.0) [27].

### Antiamastigote activity

Peritoneal macrophages from BALB/c mice were infected with promastigotes of *L. amazonensis* in Lab-Tek chambers (Nunc, Roskilde, Denmark) and kept at 37 °C. After four hours, the chambers were washed, and the cultures were treated with miconazole alone or in combination with imipramine in supplemented RPMI medium for 72 hours. Various concentrations were used for these studies, with up to 16 μM of miconazole and 50 μM of imipramine. In the combination assays, the concentration of imipramine was fixed at 12.5 μM and the concentration of miconazole varied, with the maximum concentration being 16 μM. After incubation, the slides were stained, and the infection rate was determined by counting the infected macrophages under a light microscope. The infection rate was calculated using the formula: % infected macrophages × number of amastigotes/number of total macrophages.

### Toxicity to macrophages

Mouse peritoneal macrophages in 96-well plates were treated with imipramine and miconazole alone or in combination for 72 hours at 37 °C. After this period, the cell viability was evaluated by adding 10 % tetrazolium salt (MTT) (5 mg/ml) to each well. The plates were incubated at 37 °C for another four hours, and the resulting formazan crystals were dissolved by adding 80 μl DMSO to each well. The plates were then read at 570 nm. The results were expressed as the percentage of viable cells compared to the untreated control.

### Extraction of lipids

Lipids from promastigotes of *L. amazonensis* were extracted using the method reported by Bligh & Dyer [28]. Briefly, samples were pelleted, and a solution of methanol, chloroform and water (2:1:0.5 v/v) was added. After stirring the mixture for one hour, the samples were centrifuged for 20 min at 3,000 rpm and the supernatant containing the lipids was separated from the precipitate. The precipitate was subjected to a second extraction under the same conditions. The supernatants were combined, and methanol/chloroform/water (1:0.5:0.4 v/v) was added. After 40 seconds of stirring, the material was centrifuged (3,000 rpm/30 min) again. The lower layer (organic) containing the lipids was then separated with the aid of a glass syringe and was transferred to a 1.5 ml tube resistant to organic solvents (Axygen Scientific, Inc., Union City, CA, USA). The solvent was evaporated under an N_2_ flux, and the lipids were analyzed by gas chromatography- mass spectrometry (GC/MS), as described below.

### Analysis of the sterol profile by gas chromatography coupled with mass spectrometry (GC/MS)

Promastigotes of *L. amazonensis* were cultured with 15 or 30 μM imipramine or 4 μM miconazole or in culture medium alone. After 72 hours, 1×10^8^ parasites from each culture were washed three times in cold PBS (pH 7.5), and the sterols were extracted as described in Section 3.6. The samples were injected into a GC/MS - QP2010 Ultra Machine (Shimadzu Scientific Instruments, Tokyo, Japan). After injection, the column temperature was maintained at 50 °C for one minute and then increased to 270 °C at a rate of 10 °C/min and finally to 300 °C at a rate of 1 °C/min. The flow of the carrier gas (He) was kept constant at 1.1 ml/min. The temperatures of the injector and detector were 250 °C and 280 °C, respectively [29].

#### Ethical approval

Macrophages from BALB/c mice were obtained in accordance with protocols approved by the Ethics Committee for Animal Use of the Instituto Oswaldo Cruz (L026/2015).

## Results and discussion

Imipramine has effects on the cell growth and membrane functions of *L. donovani* and *L. major* [14]*.* Antidepressants cause lethal disruption of their membrane function, reductions in mitochondrial proton motive force and apoptosis in *Leishmania* spp. [30]. In addition to the effects on the parasite itself, imipramine is also a potent inducer of TNF-α in macrophages, which is an important cytokine for antileishmanial defense [31]. Tricyclic antidepressants (imipramine, desipramine, amitriptyline and nortriptyline) are amphiphilic molecules that accumulate in membranes and alter their biophysical characteristics [16, 32]. Imipramine has also been demonstrated to inhibit the three main classes of methyltransferases (N, O and C-methyltransferases). The activity of phosphatidylethanolamine N-methyltransferase was also found to be inhibited by imipramine and chlorpromazine [33]. Moreover, imipramine has inhibitory effects on erythrocyte catechol-O-methyltransferase and indirectly inhibits DNA methylation by cytosine C-methyltransferase [17, 34].

Considering the affinity of imipramine for membranes and its effects on methyltransferases, we hypothesized that imipramine may interfere with the sterol metabolism of parasites. Therefore, we analyzed the sterol composition of promastigotes of *L. amazonensis* when they were exposed to imipramine. Table 1 shows the results of an analysis of the relative amount of each sterol. The promastigotes treated with imipramine showed a decrease in cholesterol **(1)** and in the ergostane-derived sterols (C24-methylated sterols) ergosta-5,7,24-trien-3β-ol (dehydroepisterol) **(5)** and ergosta-7,24-dien-3β-ol (episterol) **(6)**. Interestingly, we also observed a concomitant accumulation of cholestane-derived sterols (C24-demethylated sterols), cholesta-5,7,22-trien-3β-ol **(2),** cholesta-7-24-dien- 3β-ol **(3)**.

**Table 1:** Effects of imipramine and miconazole on the sterol composition of *L. amazonensis* promastigotes

		C	Imipramine 15 μM	Imipramine 30 μM	Miconazole 4 µM
Substance	PM	Relative amount (%)			
(1) Cholesterol (exogenous)	386	9.49	7,76	5,4	15.04
(2) Cholesta-5,7,22-trien-3β-ol	382	-	28,1	40,33	-
(3) Cholesta-7-24-dien- 3β-ol	384	-	2,45	4,11	-
(4) 14α-metilergosta-8,24 (24^1^)-dieno-3β-ol	412	-	-	-	46.40
(5) Ergosta-5,7,24-trien-3β-ol (dehydroepisterol)	396	83,4	56,07	48,67	4.15
(6) Ergosta-7,24-dien-3β-ol (Episterol)	398	7,1	5,62	1,49	-
(7) 4α,14α-dimetilergosta-8,24 (24^1^)-dieno-3β-ol (Obtusofoliol)	426	-	-	-	34.41

C – Untreated control

Azasterols inhibit the C24 sterol methyltransferase, an enzyme required for the production of the typical ergostane- and stigmastane-based sterols, which are not found in mammalian cells [35]. Azasterols interfere with the sterol biosynthesis of *T. cruzi* and *Leishmania* spp., resulting in cell growth arrest and death [36]. The treatment of *L. donovani* promastigotes with azasterol altered their sterol profile, leading to a global decrease in 24-methylsterols and an increase in demethylated sterols [37]. This effect is similar to that observed following the treatment with imipramine (Table 2), suggesting that it occurs *via* a similar mechanism of action.

**Table 2:** Relative proportions of 24-methylated and 24-demethylated sterols from *L. amazonensis* promastigotes treated with imipramine

	Imipramine		
	0	15 μM	30 μM
% 24-alkylated	90.51	61.69	50.16
% 24-nonalkylated	0	30.55	44.44

Various inhibitors of sterol biosynthesis (azoles, statins and allylamines) have been extensively studied in *Leishmania* spp., confirming that this pathway is an excellent target for the chemotherapy of leishmaniasis [19, 21–25, 37, 38]. As seen in Table 1, treatment with miconazole disturbs the sterol synthesis in a different fashion than imipramine, causing the accumulation of C14 methyl sterols, 14α-metilergosta-8,24 (24^1^)-dieno-3β-ol **(4)** and obtusofoliol **(7)**, sterols that typically accumulate following the inhibition of C14 demethylase [20].

**Fig. 1:**
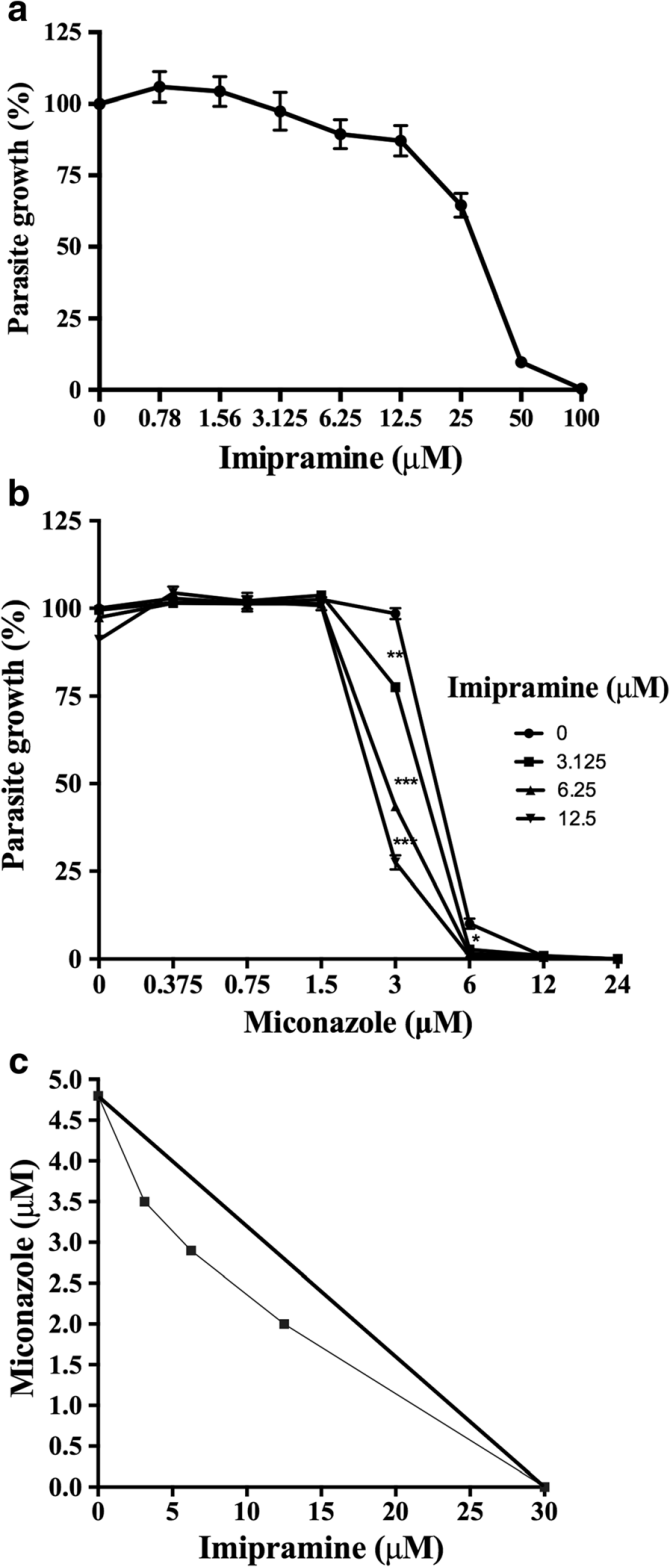
Antipromastigote activity of imipramine, miconazole and their combination. Promastigotes of *L. amazonensis* were incubated with different concentrations of imipramine with or without miconazole for 72 hours at 26 °C. **a** imipramine, **b** imipramine + miconazole **c** an isobologram. Each plotted point in the isobologram is the IC_50_ of the drugs alone or in combination. The straight line connecting the individual IC_50_values represents the theoretical line of additivity. The experiments were performed in triplicate, *n* = 3. The graphs are representative of one experiment, and the standard deviations are shown. The graphics and IC_50_ values were obtained using the GraphPad Prism 4 software. * *P* < 0.05, ** *P* < 0.01, *** *P* <0.001

Using a combination of drugs that act on different points of the same pathway has been noted to represent an interesting strategy for antimicrobial chemotherapy [20, 21]. Haughan *et al.* demonstrated that the combination of lovastatin (an HMGCoA reductase inhibitor) and miconazole (C14-demethylase inhibitor) led to synergistic activity against promastigotes of *L. amazonensis* [23]. Therefore, since both drugs (imipramine and miconazole) interfere with the sterol synthesis pathway, but generate different aberrant metabolites, we decided to evaluate the effects of their combination on the parasite growth. The antileishmanial activity of imipramine alone and in combination with miconazole in the promastigotes of *L. amazonensis* is shown in Fig. 1a and b. The effects of the combination were expressed graphically as an isobologram, where the IC_50_values of the drugs alone and in combination were plotted. The fractional inhibitory concentration index (FICI) was calculated to classify the interaction as synergistic, antagonistic or additive. As expected, miconazole was active against promastigotes, with an IC_50_ of 4.7 ± 1.03 μM. Imipramine also proved to be active alone, with an IC_50_ of 28.63 ± 1.2 μM. The combination resulted in an additive effect, as observed in the isobologram, with a FICI value of 0.83 (Fig. 1b-c), suggesting that imipramine and miconazole can be used in combination, as no antagonistic effect was observed.

**Fig. 2:**
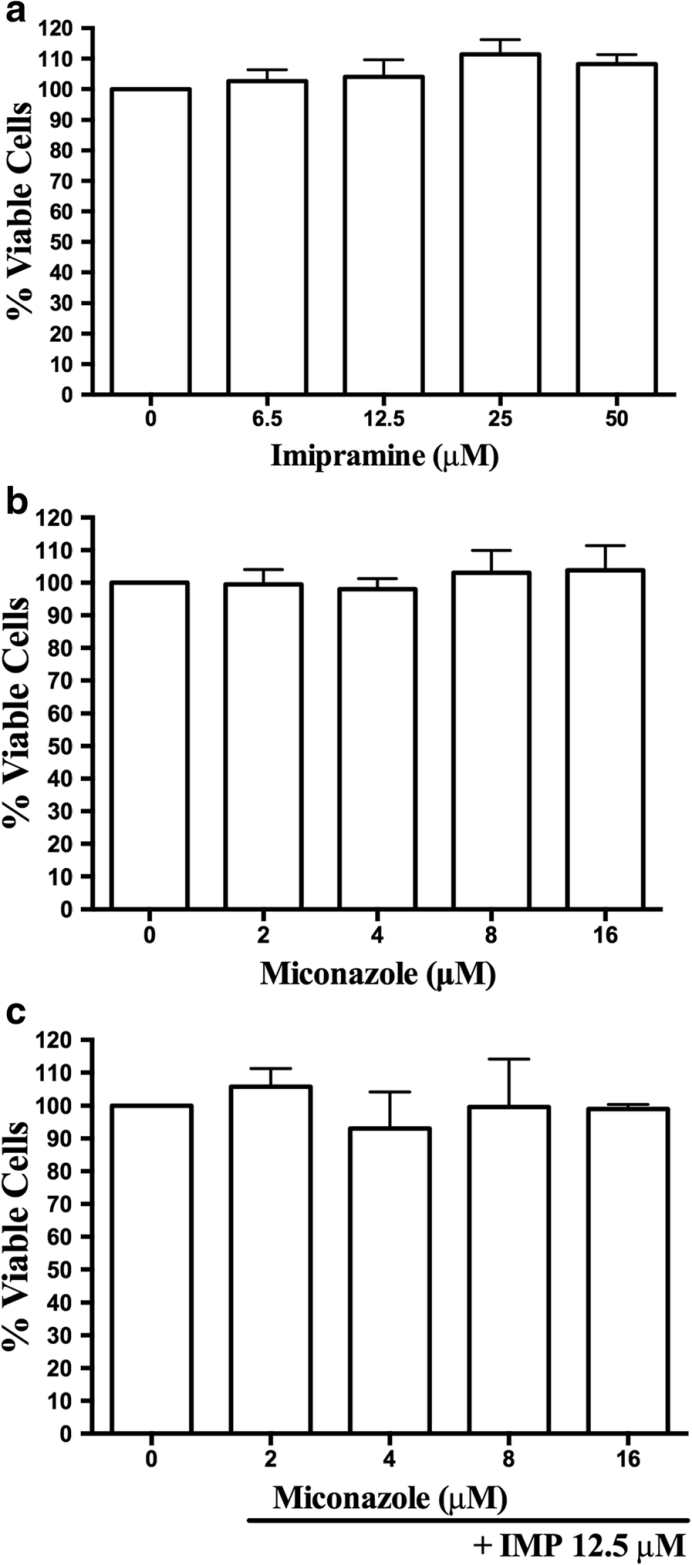
Effect on the toxicity of inhibitors peritoneal macrophages. Uninfected macrophages were incubated with imipramine, miconazole alone or the combination of the two agents for 72 hours at 37 °C. After this period, the cells were incubated with MTT for 1 hour at 37 °C, and the absorbance was read at 570 nm. **a** imipramine, **b** miconazole **c** 12.5 μM imipramine (IMP) + miconazole. The experiments were performed in triplicate, *n* = 3

Before testing the effects on intracellular amastigotes, we evaluated the toxicity of imipramine alone and in combination with miconazole in host cells. Uninfected peritoneal macrophages showed no signs of toxicity when incubated with imipramine alone at concentrations less than 50 μM (Fig. 2a). Miconazole also showed no toxic effects, even when used in combination with imipramine (Fig. 2b-c).

**Fig. 3:**
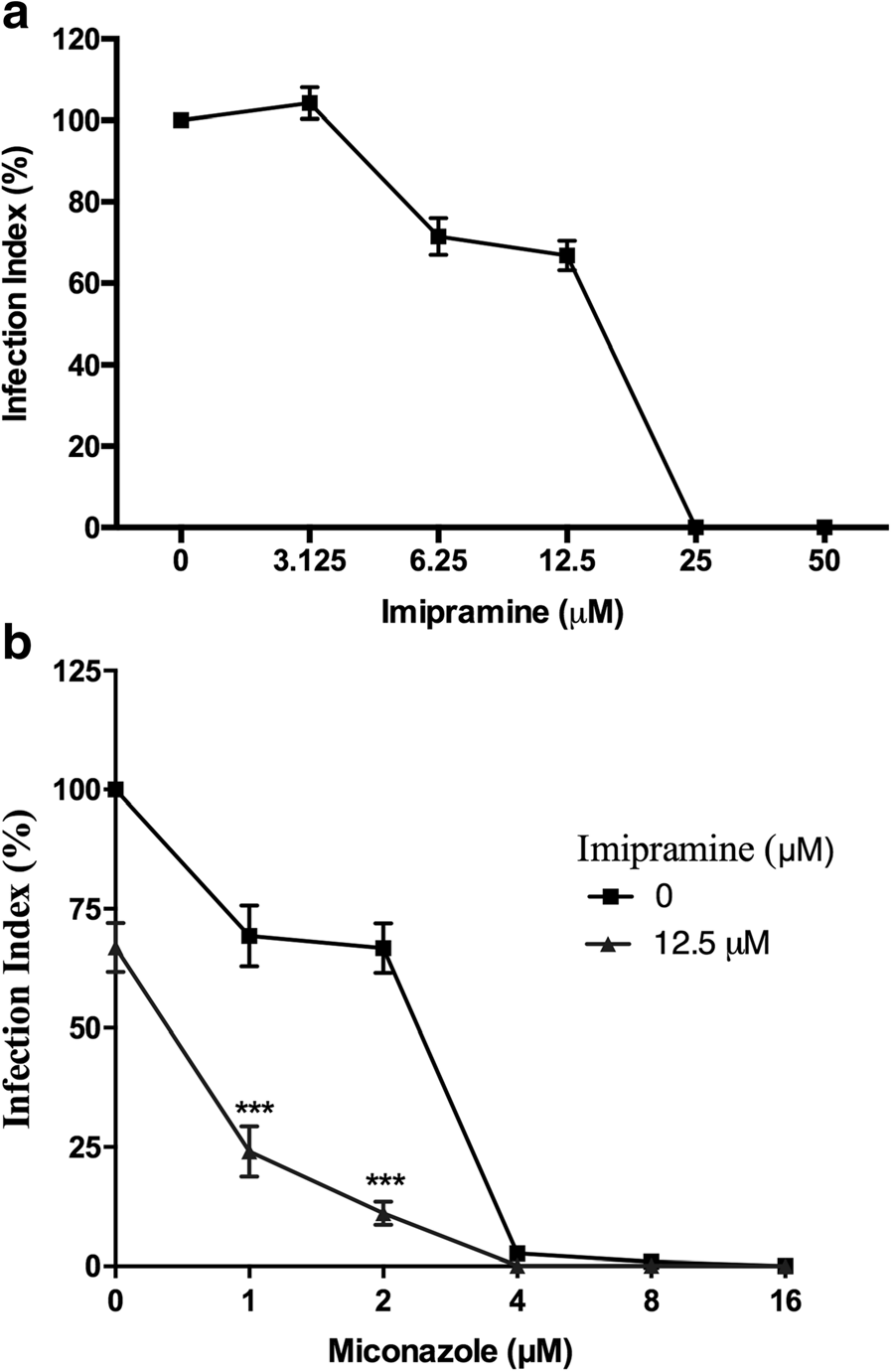
Antiamastigote activity of the combination of imipramine and miconazole. Peritoneal macrophages infected with *L. amazonensis* that were left untreated or treated with imipramine and miconazole were incubated for 72 hours at 37 °C. After incubation, the cells were fixed and stained. The infection rate was calculated using the formula: % infected MØ x number of amastigotes/total No. MØ. **a** imipramine **b** miconazole + 12.5 μM imipramine. The experiments were performed in triplicate, *n* = 3. The graphs are representative of one experiment, and the standard deviations are shown. * *P* < 0.05, ** *P* < 0.01, *** *P* < 0.001

In the next step, we assessed the activity of imipramine against intracellular amastigotes, the clinically significant stage of the parasite. The IC_50_ was found to be 13.32 ± 1.1 μM (Fig. 3a). Due to the experimental difficulty associated with performing a “checkerboard” approach to draw isobolograms for antiamastigote activity, we fixed the concentration of imipramine at 12.5 μM and varied the concentration of miconazole to calculate the IC_50_ and IC_90_ values of the combination. When used in combination with imipramine, the IC_50_ of miconazole was reduced from 2.2 ± 0.2 μM to 0.5 ± 0.1 μM and the IC_90_ was reduced from 3.9 ± 0.2 μM to 2.2 ± 0.1 μM (Fig. 3b).

In addition to having its own machinery for sterol biosynthesis, *Leishmania* also uptakes a significant amount of exogenous cholesterol, mainly through the endocytosis of LDL particles [39]. Andrade-Neto *et al.* have described that the inhibition of the sterol pathway in *L. amazonensis* increases the intake of cholesterol from the culture medium [40]. Interestingly, imipramine has been reported to block the release of cholesterol from lysosomes in mammalian cells, especially from the endocytosis of LDL particles, preventing its distribution for use by the cell [41, 42]. Therefore, it is possible that imipramine is interfering with both the biosynthesis and utilization of exogenous sources of sterols, so further studies are needed to address this issue.

## Conclusion

In summary, we have demonstrated that imipramine has antileishmanial activity, most likely by affecting the sterol biosynthesis of the parasites. The combination of imipramine with miconazole can improve the antileishmanial activity. These findings suggest that imipramine could be useful to treat leishmaniasis, particularly if used in combination with other inhibitors of sterol biosynthesis.
